# Strain effects on polycrystalline germanium thin films

**DOI:** 10.1038/s41598-021-87616-x

**Published:** 2021-04-15

**Authors:** Toshifumi Imajo, Takashi Suemasu, Kaoru Toko

**Affiliations:** 1 Institute of Applied Physics, University of Tsukuba, 1–1–1 Tennodai, Tsukuba, Ibaraki 305–8573 Japan; 2 JSPS Research Fellow, 8 Ichiban-cho, Chiyoda-ku, Tokyo, 102–8472 Japan; 3 PRESTO, Japan Science and Technology Agency, 4–1–8 Honcho, Kawaguchi, Saitama 332–0012 Japan

**Keywords:** Engineering, Electrical and electronic engineering, Materials science, Materials for devices

## Abstract

Polycrystalline Ge thin films have attracted increasing attention because their hole mobilities exceed those of single-crystal Si wafers, while the process temperature is low. In this study, we investigate the strain effects on the crystal and electrical properties of polycrystalline Ge layers formed by solid-phase crystallization at 375 °C by modulating the substrate material. The strain of the Ge layers is in the range of approximately 0.5% (tensile) to -0.5% (compressive), which reflects both thermal expansion difference between Ge and substrate and phase transition of Ge from amorphous to crystalline. For both tensile and compressive strains, a large strain provides large crystal grains with sizes of approximately 10 μm owing to growth promotion. The potential barrier height of the grain boundary strongly depends on the strain and its direction. It is increased by tensile strain and decreased by compressive strain. These findings will be useful for the design of Ge-based thin-film devices on various materials for Internet-of-things technologies.

## Introduction

Internet-of-things (IoT) will largely increase the number of electronic devices. In this regard, it is crucial to provide electronic functions to various objects. Numerous general-purpose substrates, such as low-cost glass and plastic, have low heat resistance temperatures, which limits the process temperature of device fabrication. Ge is a promising electronic material for IoT devices because of its high carrier mobilities (electron: 3900 cm^2^ V^−1^ s^−1^, hole: 1900 cm^2^ V^−1^ s^−1^) and low crystallization temperature (approximately 500 °C)^1, 2^. The performances of metal–oxide–semiconductor field-effect transistors (MOSFETs) based on a single-crystal Ge-on-insulator structure, formed using a single-crystal wafer^3–5^ and/or high-temperature process (> 900 °C)^6, 7^, surpassed those of Si MOSFETs. Low-temperature syntheses of Ge thin-film transistors (TFTs) have been achieved using conventional solid-phase crystallization (SPC)^8–11^, laser annealing^12^, seed layer technique^13^, and metal-induced crystallization^14–16^. Although these methods are potentially useful to expand the application of Ge, the crystallinity of the resulting polycrystalline Ge (poly-Ge) is still insufficient to increase the performances of Ge TFTs over those of Si MOSFETs.

Among the synthesis methods for Ge, SPC has been considerably advanced in recent years, which provided an increased grain size of poly-Ge by heat deposition of amorphous Ge (a-Ge) precursors^17^ and addition of Sn in Ge^18–20^. Accumulation-mode *p*-channel transistors based on the *p*-type Ge exhibited field-effect mobilities (170 cm^2^ V^−1^ s^−1^) higher than those of most *p*-channel Si transistors^21^. The formation of a GeO_*x*_ underlayer further improved the Hall hole mobility of poly-Ge to 620 cm^2^ V^−1^ s^−1^^22^. Although poly-Ge is generally *p*-type owing to the high hole concentration derived from defect-induced acceptors^23, 24^, *n*-type poly-Ge layers with high electron mobilities have also been achived^25, 26^.

Numerical studies have been carried out on the effects of strain in single-crystal Ge^27^, because the strain in Ge contributes to a carrier mobility increase and direct transition change^28^. Generally, poly-Ge layers have small tensile strains, although the origin of the strain has not been completely understood^9^. The strain naturally introduced in poly-Ge layers is not sufficiently large to affect the basic physical properties of Ge. However, the strain may affect the crystal growth and grain boundary (GB) properties of poly-Ge layers, which has not been extensively investigated. In this study, we control the strains in high-mobility poly-Ge layers formed by a low-temperature SPC using the difference in thermal expansion with respect to the substrate. The magnitude and direction of the strain largely influence the grain size and GB barrier height of the poly-Ge layers.

## Experimental

To modulate the strain applied to the Ge layer, we used various substrates with different coefficients of thermal expansion (CTEs), including amorphous SiO_2_, polyimide (PI), single-crystal Si (001), Ge (110), SrTiO_3_ (STO) (001), and CaF_2_ (001). We denote the CTE difference between Ge and substrate as Δ*α*; the values are presented in Table 1. Before the thin film deposition, the substrates were cleaned with acetone, methanol, and distilled water. To eliminate the difference in the interface between Ge and substrate, we fabricated 50-nm-thick GeO_*x*_ layers on the substrates using radio-frequency (RF) magnetron sputtering (base pressure: 3.0 × 10^–4^ Pa) with 10 sccm Ar plasma (working pressure: 0.5 Pa), where the RF power was set to 50 W. The sample stage was not heated or rotated. The samples were then air-transferred from the sputtering chamber to a molecular beam deposition system (base pressure: 5 × 10^−7^ Pa) within five min to avoid the reaction of GeO_*x*_ with air. Subsequently, 400-nm-thick a-Ge precursors were prepared using a Knudsen cell with a solid Ge source (purity: 99.999%) at a deposition rate of 1 nm min^−1^. During the deposition, the sample stage was heated at 150 °C to densify the a-Ge^17^. The samples were then loaded into a conventional tube furnace (Koyo Thermo Systems, KTF035N1) with N_2_ flow (purity: 99.9%, flow rate: 0.1 L min^−1^) and annealed at 375 °C for 150 h to induce SPC. The temperature was calibrated by placing a thermocouple directly on the tube furnace and was confirmed to be uniform within the sample stage.

**Table 1:** CTEs and CTE differences between Ge and substrate (Δ*α*).

Substrate	CTE [10^–6^ K^−1^]	Δ*α* [10^–6^ K^−1^]
SiO_2_	0.5	− 5.3
Si	3.9	− 1.9
Ge	5.8	0
STO	9.4	3.6
CaF_2_	18.9	13.1
PI	27.0	21.2

The resulting samples were evaluated using the Raman spectroscopy, X-ray diffraction (XRD), electron back-scatter diffraction (EBSD), and Hall effect measurement. Raman spectra were measured using a JASCO NRS-5100 with a frequency doubled Nd:YAG laser (wavelength: 532 nm, power: 0.5 mW, spot diameter: 20 μm). The objective lens magnification was 20 × . The time of acquisition for each Raman spectrum was 60 s × 2. The absolute Raman shift (detector resolution: 0.42 cm^−1^) was calibrated using the transverse optical phonon line (300 cm^−1^) of single-crystal Ge(100). Out-of-plane (*θ*–2*θ*) and in-plane (–2*θ*_χ_) XRD patterns were measured using a Rigaku SmartLab system with a Ge monochromator (wavelength: 1.54 Å) and a Cu Ka radiation source (voltage: 40 kV, current: 30 mA). The scans ranged from 20° to 60° in 0.01° steps. The EBSD analyses were performed using a JEOL JSM-7001F (voltage: 25 kV, current: 15 mA) with a TSL OIM analysis attachment. The Hall effect measurement with the Van der Pauw method was performed using a Bio-Rad HL5500PC with a 3200 G permanent magnet.

## Results and discussion

**Figure 1:**
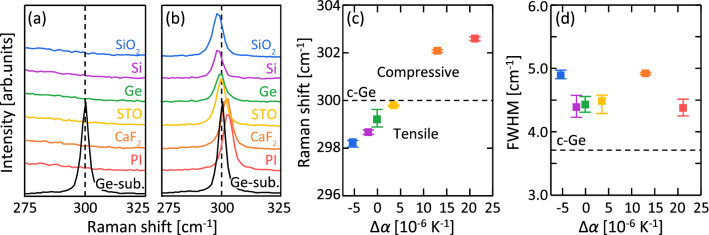
Raman spectroscopy analysis of the Ge layers on various substrates. (**a**, **b**) Raman spectra of the samples (**a**) before and (**b**) after annealing; the spectrum of a single-crystal Ge (100) wafer is shown for comparison. (**c**) Raman shifts and (**d**) FWHMs of the Ge peaks as functions of Δ*α*; the values for the Ge wafer are shown by the dotted lines. The error bars indicate the variability in the Raman measurements at five locations for each 1-cm square sample.

Raman spectroscopy and XRD are used to determine the strains and crystallinities of the Ge layers after annealing. Figure 1(a) shows that the Raman spectra of the samples before annealing have no peaks, indicating the amorphous state of Ge. Figure 1(b) shows that the peak corresponding to the Ge–Ge vibration mode is located around 300 cm^−1^, which indicates that all samples are crystallized after the annealing. The Raman shift and full width at half maximum (FWHM) of the Ge–Ge peak was analyzed from the Raman spectra. Figure 1(c) show that the Raman shift increases with Δ*α*, indicating that the strain is tensile for Δ*α* < 5 × 10^–6^ K^−1^ and compressive for Δ*α* > 5 × 10^–6^ K^−1^. Figure 1(d) show that the FWHM has no clear correlation with Δ*α*, suggesting that the Ge–Ge bond quality does not depend on Δ*α*.

**Figure 2:**
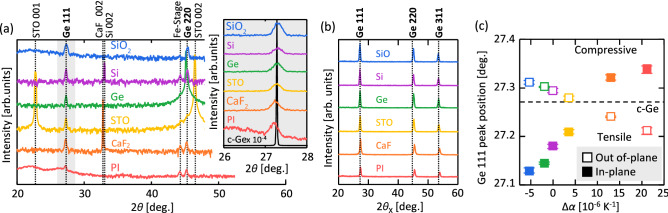
XRD analysis of the Ge layers on various substrates. (**a**) Out-of-plane *θ*–2*θ* XRD patterns; data for the bulk Ge (111) wafer are shown for comparison. The inset shows a magnified view focused on the Ge 111 peaks. (**b**) In-plane *φ*–2*θ*_χ_ XRD patterns. (**c**) Ge 111 peak position (2*θ* and 2*θ*_χ_) as a function of Δ*α*; the value for the bulk Ge (111) wafer is shown by the dotted line. The error bars indicate the variability in the XRD measurements at four locations for each 1-cm square sample.

The out-of-plane *θ*–2*θ* XRD patterns in Fig. 2(a) show that all samples exhibit diffraction peaks corresponding to the Ge 111 and 220 planes, while other large peaks appear owing to the single-crystal substrates. The inset shows that the Ge 111 peak position is slightly shifted from that of a single-crystal Ge(111) wafer and varies depending on the substrate. The in-plane *φ*–2*θ*_χ_ XRD patterns in Fig. 2(b) show Ge 111, 220, and 311 peaks for all samples. According to the Ge powder diffraction patterns (Joint Committee on Powder Diffraction Standards (JCPDS) 04–0545), both XRD results indicate that the Ge layers are polycrystalline without epitaxial relationship with the substrates owing to the amorphous GeO_*x*_ interlayer. Figure 2(c) shows that the Ge 111 peak position of the Ge layer strongly depends on Δ*α*. The peak position increases with Δ*α* in the in-plane measurement, while the opposite behavior is observed in the out-of-plane measurement. This behavior indicates that the directions of the strain in the Ge layer in the out-of-plane and in-plane measurements are opposite, which is consistent with the properties of general thin films.

**Figure 3:**
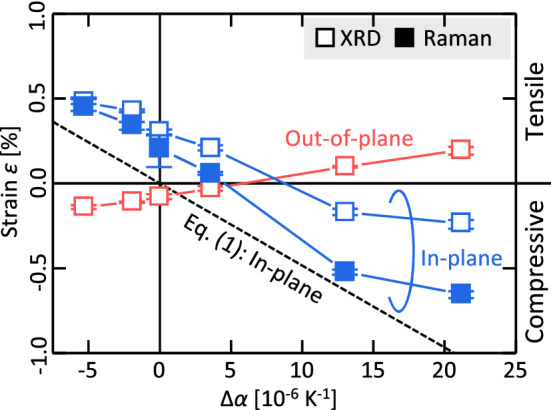
In-plane and out-of-plane strains *ε* of the Ge layer as functions of Δ*α*. The experimental data were derived from the Raman (closed squares) and XRD (open squares) measurements. The theoretical data for the in-plane direction derived from Eq. (1) are shown by the dotted line. The error bars indicate the variability in the Raman and XRD measurements for each sample.

Therefore, Δ*α* affects the strain of the Ge layer. The strain corresponding to Δ*α* should be applied to the Ge layer in the cooling process after the SPC. The strain *ε* in the thin film caused by the thermal expansion difference is expressed by^29^

1 $$\varepsilon = \frac{\Delta \alpha \cdot \Delta T}{{1 - \nu }},$$

where Δ*T* is the difference between the annealing temperature (375 °C) and room temperature (300 K) and *ν* is the Poisson’s ratio of the thin film. The in-plane *ε* in Ge attributed to the thermal expansion difference is theoretically obtained and presented by the dotted line in Fig. 3, which indicates that the Ge layer has a tensile strain for a negative Δ*α* and compressive strain for a positive Δ*α*. We estimate *ε* using the Raman shifts (Fig. 1(c)) of the Ge–Ge peak by the strain-phonon coefficient (− 395 cm^−1^) proposed by Manganelli *et al.*^30^ and Ge 111 peak positions in the XRD patterns (Fig. 2(c)) by the Bragg’s law. The *ε* values are different between the Raman and XRD analyses, which is likely because the strain-phonon coefficient varies depending on the measurement environment and sample specifications^30^. Nevertheless, *ε* obtained from the Raman and XRD analyses have similar tendency with respect to Δ*α*. The out-of-plane strain has a tendency opposite to that of the in-plane strain and exhibits smaller absolute *ε* values, which is consistent with the Poisson effect. For the in-plane direction, the slopes of experimental *ε* is almost consistent with the theoretical line derived from Eq. (1); however, the experimental *ε* values shift above the theoretical *ε* values for all Δ*α*. This suggests that the Ge layer is under a tensile stress regardless of the substrate. As known for the case of Si^31^, this occurs likely because the a-Ge tends to shrink during the crystallization owing to the density difference, while is constrained by the substrate. These results suggest that the total strain in the Ge layers is determined by not only the difference in thermal expansion with respect to the substrate, but also phase transition of Ge from amorphous to crystalline.

**Figure 4:**
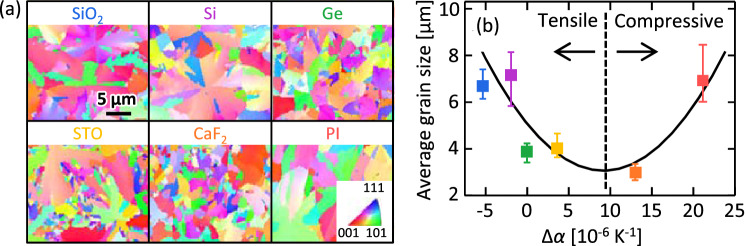
Grain sizes of the Ge layers on various substrates. (**a**) IPF images of the Ge layers, where the colors indicate the crystal orientations, according to the inset color scheme. (**b**) Average grain size determined by the EBSD analysis as a function of Δ*α*. The error bars indicate the variability in the EBSD measurements at four locations for each 1-cm square sample.

The grain size of the poly-Ge layer was evaluated using the EBSD analyses. The inverse pole figure (IPF) images in Fig. 4(a) show that all Ge layers consist of randomly oriented grains, indicating that there is no epitaxial relationship with the substrate, owing to the amorphous GeO_*x*_ underlayer. The grain sizes are large, on the order of micrometer, owing to the precursor densification and GeO_*x*_ underlayer effect^22^. The Ge grain size depends on the substrate species. Figure 4(b) shows a parabolic tendency with respect to Δ*α*. Considering the results of Fig. 3 together, Fig. 4(b) indicates that a larger |*ε*| provides a larger grain size. During the heating of the sample for SPC, the a-Ge layer also has strain owing to the CTE difference with respect to the substrate. These results suggest that the strain energy, proportional to the square of *ε*, promotes the lateral growth of Ge crystals. This behavior is consistent with the reports on strain-induced growth promotion in amorphous Si and Ge layers^14, 32, 33^.

**Figure 5:**
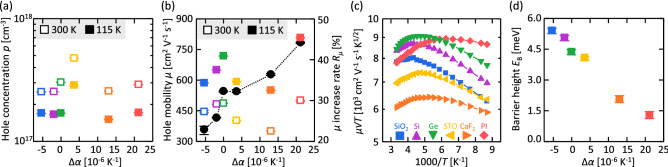
Electrical properties of the Ge layers. (**a**) Hole concentration *p* and (**b**) hole mobility *μ* measured at *T* = 115 K and 300 K as functions of Δ*α*. The *μ* increase rate *R*_*μ*_ is also shown in (**b**). (**c**) Arrhenius plots of *μT*^1/2^ for various substrates. (**d**) Grain boundary barrier height *E*_B_ as a function of Δ*α*. The error bars indicate the variability in the five measurements for each sample.

We analyze the influences of the strain on the electrical properties of the Ge layer using Hall effect measurements at 300 K and 115 K. Figure 5(a) shows that all samples exhibit hole concentrations *p* on the order of 10^17^ cm^-3^ owing to defect-induced acceptors^23, 24^. The STO sample has a relatively high *p*. Its origin is unclear. It could be attributed to the oxygen degassing from the STO substrate^34^, which may slightly damage the Ge layer. *p* is decreased for all samples at 115 K, which is a general semiconductor behavior owing to the inactivation of acceptors^24^. Figure 5(b) shows that *μ* does not have a clear tendency with respect to Δ*α*. According to the relationship between *μ* and *p* for single-crystal Ge^1^, *μ* of the studied Ge layers is limited by both impurity and GB carrier scatterings^35^. *μ* is increased for all samples at 115 K, which reflects the reduction in *p*, i.e., the impurity carrier scattering. This behavior is opposite to that of common polycrystalline semiconductors, where *μ* is limited only by GB scattering^35^. We define *μ* increase rate *R*_*μ*_ by

2 $$R_{\mu } = \frac{{\mu_{115} - \mu_{300} }}{{\mu_{300} }},$$

where the subscripts indicate the measurement temperatures (300 K and 115 K). Figure 5(b) shows that *R*_*μ*_ increases with Δ*α*. We estimate the GB barrier height *E*_B_ by the conduction model for polycrystalline semiconductors proposed by Seto^35^. According to this model, *μ* limited by GB scattering can be determined by

3 $$\mu = \frac{Lq}{{\sqrt {2\pi m^{*} kT} }}\exp \left( { - \frac{{E_{B} }}{kT}} \right),$$

where *T* is the absolute temperature, *L* is the grain size, m* is the effective mass, and *k* is the Boltzmann constant. Figure 5(c) shows that *μT*^1/2^ increases with 1000/*T*, and then begins to decrease. This behavior indicates that *μ* is affected by the impurity scattering near room temperature and limited by GB scattering at low temperatures. In addition, the shapes of the Arrhenius plots significantly differ for the different substrates, which indicates a large difference in *E*_B_. Figure 5(d) shows that *E*_B_ decreases with the increase in Δ*α*. The mechanism leading to the *E*_B_ behavior remains unclear, but it may possibly be due to the piezoelectric effect, which can occur in most polycrystalline materials^36^. The *E*_B_ behavior is responsible for the *R*_*μ*_ behavior in Fig. 5(b), i.e., the Ge layer with a larger Δ*α* has a larger influence of the impurity scattering on *μ* because of the smaller *E*_B_. Even though the GeO_2_ buffer layer is provided, it is impossible to extract only the effect of strain while using substrates of different materials. However, it was strongly suggested that the substrate that provides compressive strain is better at lowering the *E*_B_ of poly-Ge.

## Conclusions

We modulated the strain on poly-Ge formed by SPC at 375 °C using various substrates, which influenced both crystal growth and electrical properties. The strain of poly-Ge was in the range of approximately 0.5% (tensile) to -0.5% (compressive), which reflected the thermal expansion difference with respect to the substrate and phase transition of Ge from amorphous to crystalline. For both tensile and compressive strains, larger strains produced larger crystal grains, which reached sizes of approximately 10 μm. *E*_B_ was strongly dependent on the strain direction. The tensile strain increased *E*_B_, while the compressive strain reduced *E*_B_. These findings will be useful for the design of IoT devices based on poly-Ge layers on various materials.
